# Expression of recombinant *Rhipicephalus (Boophilus) microplus, R. annulatus *and *R. decoloratus *Bm86 orthologs as secreted proteins in *Pichia pastoris*

**DOI:** 10.1186/1472-6750-8-14

**Published:** 2008-02-14

**Authors:** Mario Canales, José M Pérez de la Lastra, Victoria Naranjo, Ard M Nijhof, Michelle Hope, Frans Jongejan, José de la Fuente

**Affiliations:** 1 Instituto de Investigación en Recursos Cinegéticos IREC (CSIC-UCLM-JCCM), Ronda de Toledo s/n, 13071 Ciudad Real, Spain; 2 Utrecht Centre for Tick-borne Diseases (UCTD), Department of Infectious Diseases and Immunology, Faculty of Veterinary Medicine, Utrecht University, Yalelaan 1, 3584CL, Utrecht, The Netherlands; 3 CSIRO Livestock Industries, Queensland Bioscience Precinct, 306 Carmody Road, St. Lucia, Qld 4067, Australia; 4 Department of Veterinary Tropical Diseases, Faculty of Veterinary Science, University of Pretoria, Private Bag X04, 0110, Onderstepoort, South Africa; 5 Department of Veterinary Pathobiology, Center for Veterinary Health Sciences, Oklahoma State University, Stillwater, OK 74078, USA

## Abstract

**Background:**

Rhipicephalus (Boophilus) spp. ticks economically impact on cattle production in Africa and other tropical and subtropical regions of the world. Tick vaccines constitute a cost-effective and environmentally friendly alternative to tick control. The R. microplus Bm86 protective antigen has been produced by recombinant DNA technology and shown to protect cattle against tick infestations.

**Results:**

In this study, the genes for Bm86 (*R. microplus*), Ba86 (*R. annulatus*) and Bd86 (*R. decoloratus*) were cloned and characterized from African or Asian tick strains and the recombinant proteins were secreted and purified from *P. pastoris*. The secretion of recombinant Bm86 ortholog proteins in *P. pastoris *allowed for a simple purification process rendering a final product with high recovery (35–42%) and purity (80–85%) and likely to result in a more reproducible conformation closely resembling the native protein. Rabbit immunization experiments with recombinant proteins showed immune cross-reactivity between Bm86 ortholog proteins.

**Conclusion:**

These experiments support the development and testing of vaccines containing recombinant Bm86, Ba86 and Bd86 secreted in *P. pastoris *for the control of tick infestations in Africa.

## Background

*Rhipicephalus (Boophilus) *spp. ticks are distributed in tropical and subtropical regions of the world with range expansion for some species due to changes in climatic conditions [1-3]. Infestations with the cattle tick, *Rhipicephalus (Boophilus) microplus*, economically impact cattle production by reducing weight gain and milk production, and by transmitting pathogens that cause babesiosis (*Babesia bovis *and *B. bigemina*) and anaplasmosis (*Anaplasma marginale*) [4]. *R. annulatus *and *R. decoloratus *also affect cattle production and vector pathogens in regions of Latin America, Africa or Asia [2].

Control of tick infestations has been difficult because ticks have few natural enemies. Integrated tick management strategies include the adaptation of different control methods to a geographic area. A major component of integrated tick control methods is the application of acaricides. However, use of acaricides has had limited efficacy in reducing tick infestations and is often accompanied by serious drawbacks, including the selection of acaricide-resistant ticks, environmental contamination and contamination of milk and meat products with drug residues [5]. Furthermore, development of new acaricides is a long and expensive process. All of these issues reinforce the need for alternative approaches to control tick infestations [5]. Other approaches proposed for tick control have included the use of hosts with natural resistance to ticks, pheromone-impregnated decoys for attracting and killing ticks, biological control agents and vaccines [6-8].

In the early 1990s, vaccines were developed that induced immunological protection of vertebrate hosts against tick infestations. These vaccines contained the recombinant *R. microplus *Bm86 gut antigen [8-12]. Two vaccines using recombinant Bm86 were subsequently registered in Latin American countries (Gavac) and Australia (TickGARD) during 1993–1997 [13]. These vaccines reduce the number of engorging female ticks, their weight and reproductive capacity. Thus the greatest vaccine effect was the reduction of larval infestations in subsequent generations. Vaccine controlled field trials in combination with acaricide treatments demonstrated that an integrated approach resulted in control of tick infestations while reducing the use of acaricides [12-14]. These trials demonstrated that control of ticks by vaccination has the advantages of being cost-effective, reducing environmental contamination and preventing the selection of drug resistant ticks that result from repeated acaricide application. In addition, these vaccines may also prevent or reduce transmission of pathogens by reducing tick populations and/or affecting tick vectorial capacity [13-15].

Controlled immunization trials have shown that *R. microplus *Bm86-containing vaccines also protect against related tick species, *R. annulatus *and *R. decoloratus *[16-18]. However, *R. microplus *strain-to-strain variations in the susceptibility to Bm86 vaccination have been reported, which suggests that Bm86 sequence and/or tick physiological differences may influence the efficacy of the vaccine [8,19-22]. Therefore, the cloning, expression and vaccine formulation with recombinant Bm86 from local tick strains may be required for vaccine efficacy in some geographic regions [20].

The recombinant Bm86 has been expressed in *Escherichia coli *[10], *Aspergillus nidulans *and *A. niger *[23] and *Pichia pastoris *[11,24,25]. Of these expression systems, *P. pastoris *has been shown to be the more efficient for protein secretion [26,27]. Furthermore, production of Bm86 in *P. pastoris *may increase the antigenicity and immunogenicity of the recombinant antigen [28,29]. However, the process previously reported for the production of recombinant Bm86 in *P. pastoris *is not based on protein secretion but on the expression of the antigen anchored to the yeast membrane, making necessary the purification under denaturing conditions followed by refolding of an antigen with high number of disulfide bonds [24,25,30]. Recently, *R. decoloratus *Bm86 orthologs were cloned, expressed in *E. coli *and partially characterized [31]. However, the cloning and expression of recombinant *R. annulatus *and *R. decoloratus *Bm86 orthologs in *P. pastoris *have not been reported.

The objectives of this study were (i) to clone and express in *P. pastoris *the recombinant *R. microplus*, *R. decoloratus *and *R. annulatus *Bm86 orthologs from African or Asian tick strains and (ii) to simplify the Bm86 production process by secreting recombinant proteins encoded by Bm86 orthologs in *P. pastoris*.

## Results and Discussion

### Cloning and sequence analysis of Bm86, Bd86 and Ba86

The Bm86 orthologs were cloned by RT-PCR from Mozambique *R. microplus *(Bm86), Israeli *R. annulatus *(Ba86) and South African *R. decoloratus *(Bd86) tick strains. Partial sequences were obtained and used to search the NCBI nr database for sequence identity. The first four BLAST hits (E-value = 0.0) showed that cloned Bm86, Bd86 and Ba86 sequences were identical (90–97% identity) to previously reported Bm86 (Australian Yeerongpilly reference strain; GenBank accession number M29321), Bm95 (Argentinean A strain; AF150891) and Bd86-1 and Bd86-2 (Kenyan strain; DQ630523 and DQ630524) sequences. The only fragment of 1,107 nucleotides previously reported for Ba86 (Mexican strain; AF150897) had 99.9% identity to the Ba86 sequence reported here with a single A × G substitution at position 1,674 (position 1 corresponds to the adenine in the initiation codon of the M29321 reference sequence). The Bm86 sequence of the Mozambique *R. microplus *strain reported here had a deletion of 66 nucleotides between positions 554 and 619 not found in other Bm86 sequences, which suggested that this region encoding for 22 amino acids may not be important for protein function. The Bd86 sequence of the South African *R. decoloratus *strain had an 18 nucleotides insertion between positions 1,690 and 1,691, similar to Bd86-2 and three nucleotides longer than in Bd86-1 [31].

Pairwise nucleotide and amino acid sequence alignments were conducted between cloned Bm86, Ba86 and Bd86 sequences and those identified above to have identity to these sequences (Table 1). The results showed that sequence identity was higher between Bm86 and Ba86 than with Bd86 sequences.

**Table 1:** Nucleotide and amino acid sequence comparison between Bm86 orthologs.

	*Rm *Bm86 (M29321)	*Rm *Bm95 (AF150891)	*Rm *Bm86 (EU191620)	*Ra *Ba86 (EU191621)	*Rd *Bd86-2 (DQ630524)	*Rd *Bd86-1 (DQ630523)	*Rd *Bd86 (EU191622)
	
*Rm *Bm86 (M29321)	**100**	99	94	96	90	90	90
*Rm *Bm95 (AF150891)	98	**100**	94	96	90	90	90
*Rm *Bm86 (EU191620)*	93	92	**100**	92	86	87	86
*Ra *Ba86 (EU191621)*	94	94	90	**100**	91	91	91
*Rd *Bd86-2 (DQ630524)	85	86	82	87	**100**	96	97
*Rd *Bd86-1 (DQ630523)	86	86	82	88	94	**100**	96
*Rd *Bd86 (EU191622)*	86	87	82	88	96	94	**100**

Percent identity among nucleotide (above diagonal) and percent similarity among deduced amino acid (below diagonal) sequences between Bm86 orthologs were determined. Sequences were aligned and percent identity/similarity was determined using the program AlignX. Abbreviations: *Rm, R. microplus*; *Ra, R. annulatus*; *Rd, R. decoloratus*. GenBank accession numbers are shown in parenthesis. The sequences reported in this study are identified wth an asterisk.

### Production and characterization of *P. pastoris *strains for the expression of recombinant Bm86, Bd86 and Ba86

The plasmids pPAMoz9, pPADec8 and pBaI were transformed into *P. pastoris *strains GS115, KM71H and X33 for expression of recombinant Bm86, Bd86 and Ba86 proteins. Single colonies of *P. pastoris *transformants for each gene were grown in an orbital shaker under induction conditions. Culture supernatants were spotted on a nitrocellulose membrane for dot-blot analysis of recombinant proteins. Expression of Bm86 and Bd86 was obtained in GS115 and KM71H strains while Ba86 was expressed in strain X33 only (Table 2). Expression levels varied between 1.0 and 6.0 mg·L^-1^, representing 1.5% to 13.2% of total proteins in the supernatant (Table 2). For recombinant Bm86 and Bd86, differences in expression levels were not observed between GS115 and KM71H strains. The highest expression levels were obtained for Ba86 in strain X33 (Table 2). The recombinant strains GS115Moz9-2, KM71HDec8-1 and X33pBaI-3 with highest expression levels of Bm86, Bd86 and Ba86, respectively, were selected for fermentation scale up in a 5-L bioreactor.

**Table 2:** Screening for Bm86, Bd86 and Ba86 expression in the culture supernatant of *P. pastoris *transformants.

**Recombinant strain**	**Parental strain**	**Recombinant protein**	**Total protein concentration (mg·L^-1^)^a^**	**Recombinant protein concentration (mg·L^-1^)^b^**	**% of total protein**^c^
GS115Moz9-1	GS115	Bm86	66.5	3.0	4.5
GS115Moz9-2*	GS115	Bm86	65.5	3.3	5.0
GS115Moz9-3	GS115	Bm86	65.3	1.0	1.5
KM71HMoz9-1	KM71H	Bm86	66.3	1.0	1.5
KM71HMoz9-2	KM71H	Bm86	66.8	3.0	4.5
KM71HMoz9-3	KM71H	Bm86	64.8	1.5	2.3

GS115Dec8-1	GS115	Bd86	64.4	1.0	1.6
GS115Dec8-2	GS115	Bd86	66.4	1.5	2.3
GS115Dec8-3	GS115	Bd86	66.0	1.5	2.3
KM71HDec8-1*	KM71H	Bd86	66.0	2.0	3.0
KM71HDec8-2	KM71H	Bd86	63.4	1.5	2.4
KM71HDec8-3	KM71H	Bd86	63.5	1.0	1.6

X33pBaI 1	X33	Ba86	49.7	1.0	2.0
X33pBaI 2	X33	Ba86	45.5	1.0	2.2
X33pBaI 3*	X33	Ba86	45.4	6.0	13.2
X33pBaII 1	X33	Ba86	55.8	5.5	9.8
X33pBaII 2	X33	Ba86	48.3	5.0	10.4
X33pBaII 3	X33	Ba86	46.9	4.0	8.5

The experiments were conducted twice with similar results.

^a^Determined using the Bradford method with BSA as standard [49].

^b^Determined by semi-quantitative analysis in dot-blots using a standard curve constructed with known amounts of recombinant Bm86 extracted from Gavac (Revetmex).

^c^Determined as the percent of recombinant protein in total preoteins.

*Recombinant strains with highest Bm86, Bd86 and Ba86 concentration in the culture supernatant were selected for fermentation and protein production.

The GS115Moz9-2, KM71HDec8-1 and X33pBaI-3 high expression strains had a Mut^S ^phenotype (Table 3). It has been demonstrated that transformation of *P. pastoris *with plasmids using the *AOX1 *expression system may lead to three mutant phenotypes with regard to methanol utilization [32]. The Mut^+ ^phenotype grows on methanol at the wild-type rate and requires high feeding rates of methanol, the Mut^S ^phenotype has a disruption in the *AOX1 *gene and has a slower specific growth rate in methanol and the Mut^- ^is unable to grow in methanol. Although transformation of X-33 and GS115 strains with linearized constructs favor single crossover recombination at the *AOX1 *locus and generates a Mut^+ ^phenotype, double crossover recombination that results in the disruption of the wild-type *AOX1 *gene and the generation of a Mut^S ^phenotype is possible. The *P. pastoris *strains with a Mut^S ^phenotype grow slower in methanol but may be better hosts for the secretion of recombinant proteins [33].

**Table 3:** Characterization of the fermentation process for the secretion of recombinant Bm86, Bd86 and Ba86.

**Recombinant strain**	**Mut phenotype**	**O.D. _600 nm _before induction**	**μ_max _in glycerol (h^-1^)^a^**	**μ_max _in methanol (h^-1^)^b^**	**Total protein concentration (mg·L^-1^)^c^**	**Recombinant protein**		
						
						**Concentration (mg·L**^-1^**)**^c^	**Purity (%)**^c^	**Productivity (mg·L**^-1^**·h**^-1^**)**^c^
GS115Moz9-2	Mut^S^	115	0.181	0.005	274	150	55	2.1
KM71HDec8-1	Mut^S^	125	0.182	0.002	194	110	57	1.5
X33pBaI 3	Mut^S^	125	0.178	0.003	170	112	66	1.6

^a^The maximum growth rate (μ_max_) was determined during the exponential growth phase on glycerol in batch and fedbatch modes.

^b^The maximum growth rate (μ_max_) was determined during the exponential growth phase on methanol (first 20–24 hrs after induction).

^c^Determined in the culture medium 72 hrs after induction with methanol using the Bradford method with BSA as standard [49] and the Experion semiautomated electrophoresis system (Bio-Rad, Hercules, CA, USA).

### Expression of recombinant Ba86, Bd86 and Bm86 proteins in *P. pastoris*

The GS115Moz9-2, KM71HDec8-1 and X33pBaI-3 strains were used for bench-top fermentation exploiting the methanol utilization ability of *P. pastoris *strains in PM medium. This medium was previously used for *P. pastoris *fermentations to express high levels of recombinant Bm86 [24,34].

The initial phase of the fermentation process (biomass production phase) ended after 20–24 hrs and induction of recombinant protein expression started at the onset of methanol-adoption and utilization phases. As expected, all strains behaved similarly when growing on glycerol as the sole carbon source (Table 3). Cell densities before induction and maximum growth rates on glycerol were very similar and similar to those previously reported in *P. pastoris *[33,35].

**Figure 1:**
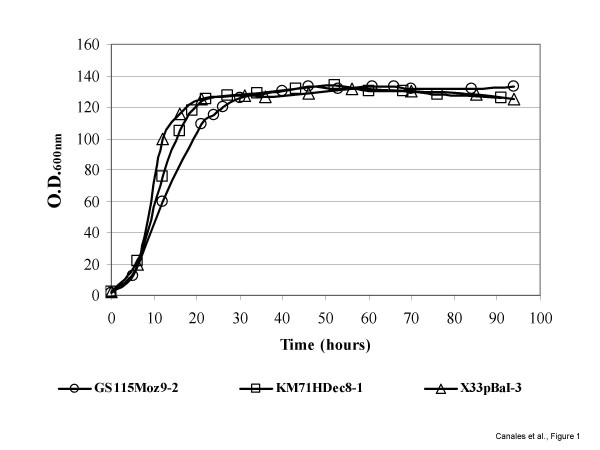
**Characterization of the growth of *P. pastoris *strains during the fermentation process**. Time profile of optical density measurements of *P. pastoris *strains GS115Moz9-2, KM71HDec8-1 and X33pBaI-3 expressing recombinant Bm86, Bd86 and Ba86, respectively.

**Figure 2:**
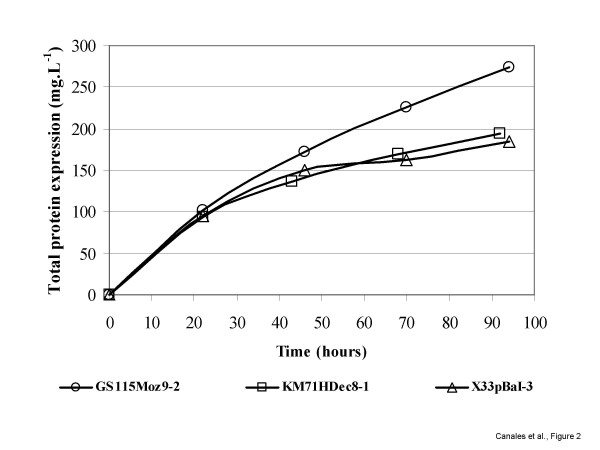
**Characterization of protein secretion in *P. pastoris *strains during the fermentation process**. Time profile of total protein concentration in the culture medium of *P. pastoris *strains GS115Moz9-2, KM71HDec8-1 and X33pBaI-3 expressing recombinant Bm86, Bd86 and Ba86, respectively.

The selected fed-batch strategy to feed methanol was identical for all strains. Once glycerol used as carbon source in the initial batch and fed-batch phases was consumed, recombinant protein expression was induced by the addition of methanol to the culture medium. An exponential growth phase was then observed during the next 20–24 hrs with maximum growth rates of 0.005, 0.002 and 0.003 h^-1 ^for the strains GS115Moz9-2, KM71HDec8-1 and X33pBaI-3, respectively. However, after 24 hrs growth in methanol, cells stop growing and a steady increase in pO_2 _levels revealed that a stationary growth phase was achieved. Nevertheless, total protein production continued to increase gradually to 274, 194 and 170 mg·L^-1 ^for the strains GS115Moz9-2, KM71HDec8-1 and X33pBaI-3, respectively (Table 3 and Figs. 1 and 2).

In this first approach to obtain recombinant Bm86, Bd86 and Ba86 secreted to the culture medium, methanol was supplied at 1 ml·h^-1^·L of the initial fermentation volume for the first two hrs and then methanol supply was increased in 10% increments every 30 min to a rate of 3 ml·h^-1^·L. This strategy probably did not allow maintaining a steady concentration of methanol throughout the whole fermentation process and either starvation or accumulation of methanol could have occurred. This fact may explain lower growth rates and expression levels of recombinant Bm86, Bd86 and Ba86 when compared to the 65 g·L^-1 ^dry weight and 1.5 g·L^-1 ^of recombinant protein previously reported for membrane-bound Bm86 in *P. pastoris *[11,24,34]. These results suggest that recombinant Bm86, Bd86 and Ba86 protein expression levels may be increased by the optimization of the fermentation and methanol induction processes.

**Figure 3:**
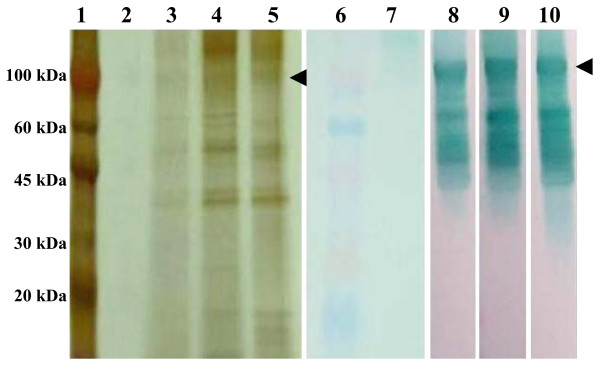
**Secretion of recombinant Bm86, Bd86 and Ba86 by *P. pastoris***. Silver stained SDS-PAGE (lanes 1–5) and Western blot analysis (lanes 6–10) of the fermentation culture supernatants after 72 hrs growing in methanol. Samples of 15 μL were loaded in each well. Membranes for Western blot were probed with serum from rabbits immunized with control Bm86 (Gavac; Revetmex) diluted 1:1000. Membranes were then washed three times with TBS and incubated with an anti-rabbit IgG HRP conjugate (Sigma-Aldrich) diluted 1:1000 in TBS. After washing the membranes again, color was developed using TMB stabilized substrate for HRP (Promega). Lanes 1 and 6: molecular weight markers (MW; ColorBurst, Sigma-Aldrich). Lanes 2 and 7: culture supernatants of the *P. pastoris *GS115/Albumin negative control strain. Lanes 3 and 8, 4 and 9, and 5 and 10: culture supernatants of X33pBaI-3 (Ba86), GS115Moz9-2 (Bm86) and KM71HDec8-1 (Bd86) strains, respectively. The position of recombinant proteins is indicated with arrows.

The presence of recombinant proteins in the culture supernatant was demonstrated at the end of the fermentation process by SDS-PAGE and Western blot (Fig. 3). Recombinant Bm86, Bd86 and Ba86 secreted in *P. pastoris *appeared in SDS-PAGE and Western blots as a major wide band with a size range of 100 to 110 kDa and smaller degradation fragments (Fig. 3). The recombinant Bm86 previously expressed in *P. pastoris *also had degradation products and a major wide band, but with a size ranging from 90 to 100 kDa [11]. These differences in estimated molecular weight of the proteins may be due to strain differences in glycosylation, which is responsible for the wide appearance of the protein band in the SDS-PAGE and Western blot [11].

### Protein recovery and purification

To obtain a clarified supernatant for recombinant protein purification, a primary centrifugation step was performed at 3,900 × g. Due to the fact that *P. pastoris *culture centrifugation at g-forces between 3,000–5,000 results in a significant product entrainment [36], a washing step of cell pellets was made for the full recovery of secreted proteins.

*P. pastoris *secretes few autologous proteins [37]. Therefore, heterologous protein secretion serves as the major first step in recombinant protein purification. However, unclear supernatants and recombinant protein purities ranging between 55% and 66% suggested the presence of contaminants in the supernatant after cell separation (Table 4). This observation suggested that probably cell lysis occurred during the stationary phase of the fermentation process due to suboptimal growth conditions. Cell lysis during the fermentation may have contributed to protein degradation, thus affecting recombinant protein yield and reinforcing the need for optimization of the fermentation process to reduce protein degradation and increase expression levels.

**Table 4:** Characterization of the recombinant Bm86, Bd86 and Ba86 purification process.

**Purification stages**	**Bm86**				**Bd86**				**Ba86**			
	Total protein conc. (mg·L^-1^)	Rec. protein conc. (mg·L^-1^)	Purity (%)	Recovery (%)	Total protein conc. (mg·L^-1^)	Rec. protein conc. (mg·L^-1^)	Purity (%)	Recovery (%)	Total protein conc. (mg·L^-1^)	Rec. protein conc. (mg·L^-1^)	Purity (%)	Recovery (%)
Fermentation supernatant	274	150	55	---	194	110	57	---	170	112	66	---
Culture separation and microfiltration	137	96	70	60	84	63	75	55	99	77	78	62
Ultrafiltration and diafiltration	407	326	80	35	451	370	82	42	370	314	85	40

Abbreviations: conc., concentration; rec., recombinant.

**Figure 4:**
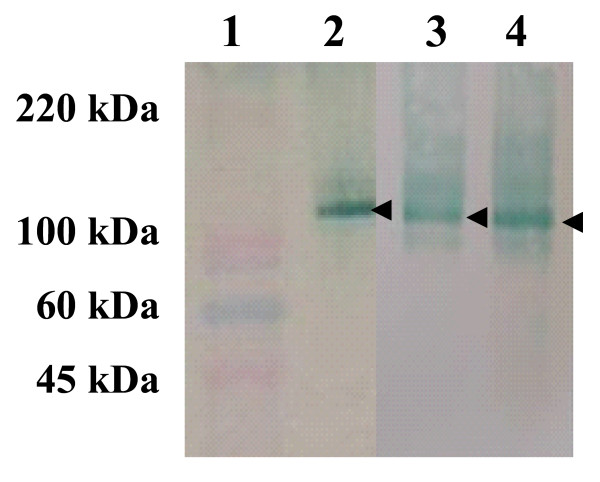
**Characterization of purified recombinant proteins**. Western blot analysis of the purified recombinant Bm86 (lane 2), Bd86 (lane 3) and Ba86 (lane 4) proteins. On each well, 3.5 μg proteins were loaded. Membranes were probed with serum from rabbits immunized with control Bm86 (Gavac; Revetmex) diluted 1:1000. Membranes were then washed three times with TBS and incubated with an anti-rabbit IgG HRP conjugate (Sigma-Aldrich) diluted 1:1000 in TBS. After washing the membranes again, color was developed using TMB stabilized substrate for HRP (Promega). Lane 1: molecular weight markers (MW; ColorBurst, Sigma-Aldrich). The position of recombinant proteins is indicated with arrows.

It has been demonstrated in previous cell fractionation experiments of *P. pastoris *that a wide range of particles densities and sizes are present in a disrupted suspension of the yeast [38,39]. Therefore, to separate particles in suspension from secreted recombinant proteins, supernatants were filtered throughout 5, 0.45 and 0.22 μm filtration systems, which resulted in 20–25% increase in recombinant protein purity (Table 4). Finally, size exclusion and diafiltration through a 50 kDa cut-off membrane resulted in 80–85% pure recombinant proteins (Table 4 and Fig. 4).

The purity of recombinant proteins reported herein after protein secretion and a simple centrifugation-filtration purification process was higher than that obtained for membrane-bound Bm86 [24,34]. The purification of the membrane-bound Bm86 required cell disruption, washing of cell pellet, denaturation, renaturation and protein precipitation procedures [24,34]. In spite of the high level expression obtained during fermentation [11,34] and the optimization of the purification process [24,40-43] for the membrane-bound Bm86, the secretion of recombinant Bm86 in *P. pastoris *reported herein allowed for higher recovery and purity of recombinant protein after purification.

Additionally, an important advantage of secreting recombinant proteins in *P. pastoris*, particularly for proteins with complex structures and a high number of disulfide bonds such as Bm86 [44], is that the isolation of a membrane-bound form under denaturing conditions followed by refolding is very unlikely to reform all disulfide bonds correctly and reproducibly. By contrast, if disulfide bond formation occurs through the natural cell processing and secretion machinery as reported herein, the product is more likely to have a reproducible conformation closely resembling the native protein.

The recombinant Bm86 has been expressed in *E. coli *[10], *A. nidulans *and *A. niger *[23] and *P. pastoris *[11,24,25]. Other expression systems using arthropod cell lines have been considered. However, despite recent advances in the application of insect cell culture technology for the production of recombinant proteins, the process is still more expensive and difficult to scale-up when compared to proteins expressed in *E. coli *and *P. pastoris *[45]. The secretion of recombinant Bm86 ortholog proteins reported here in *P. pastoris *is easy to scale-up, simple, reproducible and likely to result in a product with high antigenicity and immunogenicity [28,29].

### Characterization of recombinant Bm86, Bd86 and Ba86

Although differences may exist in antigen recognition between cattle and rabbits [46], rabbits have been proven to recognize some Bm86 protective epitopes [11,47] and were therefore considered a suitable host to evaluate immune cross-reactivity between recombinant Bm86 ortholog proteins.

**Figure 5:**
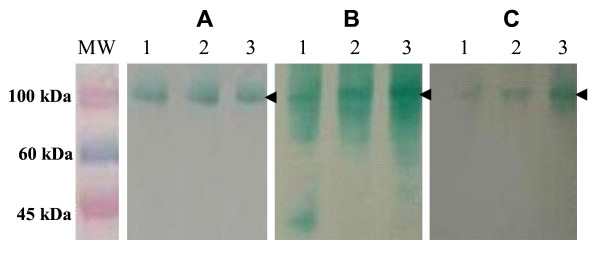
**Immune cross-reactivity between Bm86 ortholog proteins**. Western blot analysis of the purified recombinant Ba86 (lane 1), Bd86 (lane 2) and Bm86 (lane 3) proteins. On each well 1.5 μg proteins were loaded. Membranes were probed with serum from rabbitts immunized with recombinant Ba86 (A), Bd86 (B) and Bm86 (C) diluted 1:5000. Membranes were washed three times with TBS and incubated with an anti-rabbit IgG HRP conjugate (Sigma-Aldrich) diluted 1:1000 in TBS. After washing the membrane again, color were developed using TMB stabilized substrate for HRP (Promega). MW: molecular weight marker (ColorBurst, Sigma-Aldrich). The position of recombinant proteins is indicated with arrows.

The purified recombinant Bm86, Bd86 and Ba86 were adjuvated and used to immunize rabbits. The sera from immune rabbits were used to evaluate by Western blot the immune cross-reactivity between Bm86 ortholog proteins. The results showed that recombinant Bm86, Bd86 and Ba86 contained cross-reactive epitopes (Fig. 5). These results are in agreement with previous reports for Bd86 [31] and may explain, at least in part, the efficacy of the Bm86-containing vaccine against *R. annulatus *and *R. decoloratus *infestations [16-18]. However, despite immune cross-reactivity between Bm86 ortholog proteins, the differences in the efficacy of Bm86-containing vaccines against different *Rhipicephalus *spp. may be attributed to differences in the sequence of protective epitopes and/or physiological differences between tick species. Only cattle vaccination experiments with the recombinant antigens obtained here and challenging with homologous and heterologous *Rhipicephalus *spp. could fully address this question.

## Conclusion

We have cloned and secreted in *P. pastoris *the recombinant *R. microplus*, *R. decoloratus *and *R. annulatus *Bm86 orthologs from African or Asian tick strains. To our knowledge, this is the first study of Bm86, Bd86 and Ba86 secretion in *P. pastoris*. The results reported herein have shown that in *P. pastoris*, Bm86 ortholog recombinant proteins are secreted and purified from the culture supernatant with high yield and purity. The purification process for secreted proteins was simpler than that described for membrane-bound Bm86, which suggests the possibility of simplifying the purification process for recombinant Bm86 when secreted in *P. pastoris*. Additionally, secretion of recombinant Bm86 ortholog proteins in *P. pastoris *is likely to result in a more reproducible conformation closely resembling the native protein. Finally, the preliminary immunological characterization of recombinant Bm86, Bd86 and Ba86 evidenced the presence of cross-reactive epitopes among these proteins. These results suggest that these recombinant antigens can be used for the development of vaccines for the control of tick infestations in Africa. The control of livestock *Rhipicephalus *spp. infestations in Africa would contribute to improve animal health and production in this region.

## Methods

### Media and solutions

All reagents used in this work were purchased from Sigma-Aldrich (St Louis, MO, USA) or VWR International Eurolab S.L. (Mollet del Vallés, Barcelona, Spain). The compositions of the media used in this study were as follows:

Minimal methanol medium (MM): 13.4 g·L^-1 ^yeast nitrogen base with ammonium sulphate and without amino acids (YNB); 0.0004 g·L^-1^biotin; 15 g·L^-1 ^agar and 0.5% methanol.

Minimal methanol medium + Histidine (MMH): 13.4 g·L^-1 ^YNB; 0.0004 g·L^-1 ^biotin; 15 g·L^-1 ^agar; 0.04 g·L^-1 ^histidine and 0.5% methanol.

Minimal dextrose medium (MD): 13.4 g·L^-1 ^YNB; 0.0004 g·L^-1 ^biotin; 15 g·L^-1 ^agar and 20 g·L^-1 ^dextrose.

Minimal dextrose medium + Histidine (MDH): 13.4 g·L^-1 ^YNB; 0.0004 g·L^-1 ^biotin; 15 g·L^-1 ^agar; 20 g·L^-1 ^dextrose and 20 g·L^-1 ^dextrose.

Yeast Extract Peptone medium (YP): 10 g·L^-1 ^yeast extract and 20 g·L^-1 ^peptone.

Yeast Extract Peptone Dextrose medium (YPD): 10 g·L^-1 ^yeast extract; 20 g·L^-1 ^peptone and 20 g·L^-1 ^glucose.

Yeast Extract Peptone Dextrose Sorbitol medium (YPDS): 10 g·L^-1^yeast extract; 20 g·L^-1 ^peptone; 20 g·L^-1 ^glucose; 182 g·L^-1 ^sorbitol and 20 g·L^-1 ^agar.

Trace element solution (TES): 2.0 g·L^-1 ^ZnSO_4 _× 7H_2_O; 0.02 g·L^-1 ^CuSO_4 _× 5H_2_O; 0.08 g·L^-1 ^KI; 0.3 g·L^-1 ^MnSO_4 _× H_2_O; 0.19 g·L^-1^Na_2_MoO_4 _× H_2_O; 0.02 g·L^-1 ^H_3_BO_3 _and 2.9 g·L^-1 ^FeCl_3_.

Vitamin solution (VT): 0.4 g·L^-1 ^calcium pantothenate; 0.4 g·L^-1 ^tyamine; 4 g·L^-1 ^myo-inositol; 0.1 g·L^-1 ^nicotinic acid; 0.4 g·L^-1 ^pyridoxine and 0.4 g·L^-1 ^biotin.

Production medium (PM): 13 g·L^-1 ^KH_2_PO_4_; 8.75 g·L^-1 ^(NH_4_)_2_SO_4_; 4.5 g·L^-1 ^MgSO_4_; 0.5 g·L^-1 ^CaCl_2 _× 2H_2_O; 2.5 g·L^-1 ^yeast extract; 5 ml·L^-1 ^TES and 5 ml·L^-1 ^VT.

### Cloning of *R. microplus*, *R. annulatus *and *R. decoloratus *Bm86 orthologs and sequence analysis

Tick strains were obtained from laboratory colonies maintained at the Utrecht Centre for Tick-borne Diseases, Department of Infectious Diseases and Immunology, Faculty of Veterinary Medicine, Utrecht University, Utrecht, The Netherlands. Originally, tick strains were collected from infested cattle in Mozambique (*R. microplus*), Israel (*R. annulatus*) and South Africa (*R. decoloratus*).

Total RNA was extracted from the viscera of partially fed *R. annulatus *and *R. microplus *females and from eggs of *R. annulatus *and *R. decoloratus *using TriReagent (Sigma-Aldrich, St Louis, MO, USA) and following manufacturer's recommendations. Bm86 (*R. microplus*), Ba86 (*R. annulatus*) and Bd86 (*R. decoloratus*) coding regions (nucleotides 58–1884 of the coding region of Bm86 reference sequence; GenBank accession number M29321) lacking the signal peptide and GPI anchor sequences were amplified by RT-PCR. The RT-PCR was done using 10 pmol of each primer (CZABM5: 5'-A CTC GAG AAA AGA GAG TCA TCC ATT TGC TCT GAC TTC GG and CZABM3: 5'-A TCT AGA TTA AGC ACT TGA CTT TCC AGG ATC TG; Bm86 homologous regions are underlined) in a 50-μl volume (1.5 mM MgSO_4_, 1 × avian myeloblastosis virus (AMV) RT/*Thermus flavus *(*Tfl*) reaction buffer, 0.2 mM each deoxynucleoside triphosphate (dNTP), 5 u AMV RT, 5 u *Tfl *DNA polymerase) employing the Access RT-PCR system (Promega, Madison, WI, USA). Reactions were performed in an automated DNA thermal cycler (Techne model TC-512, Cambridge, England, UK). RNA was reverse transcribed for 45 min at 45°C prior to PCR consisting of an initial step of 2 min at 94°C followed by 35 cycles of a denaturing step of 30 sec at 94°C and an annealing-extension step of 2 min at 68°C. Control reactions were done using the same procedures, but without RNA added to control contamination of the PCR. PCR products were electrophoresed on 1% agarose gels to check the size of amplified fragments by comparison to a DNA molecular weight marker (1 Kb Plus DNA Ladder, Promega). The amplicon was resin purified (Wizard, Promega) and cloned into pGEM-T vector (Promega). Partial sequences of cloned Bm86 orthologs were obtained by double-stranded dye-termination cycle sequencing (Core Sequencing Facility, Department of Biochemistry and Molecular Biology, Noble Research Center, Oklahoma State University and Secugen S.L, Madrid, Spain). At least three clones from independent PCR reactions were sequenced for each gene. Multiple sequence alignment was performed using the program AlignX (Vector NTI Suite V 8.0, InforMax, Invitrogen, Carlsbad, CA, USA) with an engine based on the Clustal W algorithm [48]. Searches for sequence similarity were performed at the ncbi with the BLASTN program against the nonredundant sequence database nr.

The GenBank accession numbers for Bm86 (*R. microplus*), Ba86 (*R. annulatus*) and Bd86 (*R. decoloratus*) are EU191620–EU191622.

### Construction of expression plasmids

Bm86, Ba86 and Bd86 coding regions were excised from pGEM-T by *Xho *I and *Xba *I digestion (restriction sites introduced during PCR by CZABM5 and CZABM3 primers, respectively) and cloned into *P. pastoris *expression vector pPICZαA (Invitrogen) digested with *Xba *I and *Xho *I. In this way, Bm86 orthologs were cloned under the control of the alcohol oxidase (*AOX1*) promoter, in frame with the yeast alfa-factor secretion signal but without the C-terminal c-myc/His tag due to a translation termination site introduced by CZABM3 primer during PCR. The expression constructs were sequenced at both ends and selected constructs with correct sequences were named pPAMoz9 (Bm86), pPADec8 (Bd86) and pBaI (Ba86) and used for transformation of *P. pastoris*.

### *Pichia pastoris *transformation and screening for recombinant protein expression

Expression plasmids were linearized by restriction with *Sac *I and transformed into *P. pastoris *strains GS115, KM71H and X33 (Invitrogen) by electroporation as described [49]. Transformants were selected on YPDS plates containing 100 μg·ml^-1 ^Zeocin and incubated at 30°C. A functional assay to directly screen for high expression recombinant clones was made by culturing the transformants in an orbital shaker at 250 rpm and 30°C. Single colonies were inoculated in 1 ml YPDS containing 100 μg·ml^-1 ^Zeocin and grown overnight. Cultures were divided into two parts of 500 μl each. Five hundred μl were transferred to 5 ml fresh YP medium with 20 g·L^-1 ^glycerol, grown for 24 hrs and inoculated into 250 ml fresh YP medium supplemented with 20 g·L^-1 ^glycerol. Growth in glycerol was resumed after 24 hrs and then methanol was added daily to 1% (v/v) during the course of induction. After 5 days growing on methanol, supernatants were collected by centrifugation for 15 min at 15,000 × g in a Beckman Allegra™ X-22R centrifuge, rotor F2402H (Beckman-Coulter, Palo Alto, CA, USA) and dot blots were made to screen for expression of recombinant proteins. The other 500 μl were also transferred to 5 ml fresh YP medium with 20 g·L^-1 ^glycerol, grown for 24 hrs and mixed with glycerol to 250 g·L^-1^. Long term stocks were prepared as 100 μl aliquots and stored frozen at -80°C.

### Analysis of the Mut phenotype in *P. pastoris *transformed strains

The high expression transformants of X33 and GS115 strains were analyzed for Mut^+ ^or Mut^S ^phenotype using the functional assay described in the Invitrogen user's manuals K1710-01 and K1750-01 [49]. The KM71H strain always produces a Mut^S ^phenotype [49]. Briefly, 50 μl from the long term stocks of the high expression X33 and GS115 transformants were streaked in YPDS plates containing 100 μg·ml^-1 ^Zeocin and incubated at 30°C for 24 hrs. One colony of each transformant was streaked in both MMH and MDH plates for the GS115 and X33 strains. To differentiate Mut^+ ^from Mut^S^, control GS115/Albumin (Mut^S^) and GS115/pPicz/lacZ (Mut^+^) strains (Invitrogen) were streaked in the MMH and MDH plates. Plates were incubated at 30°C for 3 days and cell growth was observed and compared to controls.

### Fermentation process

Pre-inoculums and inoculums for bioreactor cultures were grown in a shaker at 30°C and 250 rpm. Two 100 μl long term stock vials were seeded in 1 ml YP medium, grown for 12 hrs and transferred into 4 × 50 ml tubes containing 5 ml of YP medium with 20 g·L^-1 ^glycerol. After 24 hrs, cultures were mixed again and 5 ml were used to inoculate 2 L Erlenmeyer flasks containing 250 ml of YP medium with 20 g·L^-1^glycerol. Cells were grown to an O.D._600 nm _between 15 and 20 and then cultures were inoculated into a 5-L working volume Biostat B bioreactor (B. Braun Biotech, Melsungen, Germany) containing 3.5 L of PM with 40 g·L^-1 ^glycerol.

During the fermentation process, temperature was kept at 30°C and dissolved oxygen was maintained at 30% saturation by regulating agitation and aeration rates. A three-phase cultivation protocol was used in the fermentation. The glycerol growth phase included a 12 to 14 hrs batch stage from the starting point followed by a 10 to 12 hrs glycerol fed-batch stage. A glycerol solution of 50% (v/v) was added to the fermentor for 4 hrs to reach an equivalent total quantity of 60 g·L^-1 ^in the culture medium. Upon exhaustion of glycerol, indicated by a sharp increase in dissolved oxygen, methanol induction was made by adding 1% (v/v) methanol to the culture medium and 3 hrs later the fed-batch phase was started by feeding methanol according to the *P. pastoris *Fermentation Process Guideline [49]. The pH was allowed to drop to 3.5 during the whole glycerol phase and it was maintained in this value by the addition of NH_4_OH. Prior to methanol induction, pH was adjusted and maintained at 5.5 by adding NH_4_OH or H_3_PO_3_. Throughout the fermentation processes, supplements of 20 ml TES and VT solutions were added to the culture medium every 24 hrs. Additionally, GS115 strain cultures were supplemented with 0.04 g·L^-1 ^L-Histidine every 24 hrs.

### Biomass analysis during fermentation

Time-course samples were withdrawn from the fermentor at regular intervals to check growth rate and protein concentration in the supernatant. Cell density was expressed as O.D._600 nm_, either measured as grams of wet weight per litter broth (O.D._600 nm _= 1.39 × wet weight (g·L^-1^) - 27.26), which was obtained by centrifugation of the samples at 15,000 × g for 15 min or measured directly in the culture medium. Total protein concentration in the culture medium was quantified using the Bradford method with BSA as standard [50].

### Cells harvesting, recovery and purification of recombinant proteins

Cultures from the 5-L fermentor were centrifuged at 3,900 × g for 15 min in a Beckman Allegra™ X-22R centrifuge, rotor SX4250 (Beckman-Coulter) to separate cells. Supernatants were then collected and filtered through a tandem filtration system with a 20 μm cartridge (Sartorius AG, Goettingen, Germany), 5 μm and 0.45-0.22 μm cartridges (Millipore, Billerica, MA, USA) and checked for total and recombinant protein content using the Bradford method with BSA as standard [50] and the Experion semiautomated electrophoresis system (Bio-Rad, Hercules, CA, USA). For the Experion, 4 μl of denatured proteins were loaded into a Pro 260 Chip and protein concentration was determined following manufacturer's recommendations. Recombinant proteins were separated by size exclusion using a Sartocon^® ^Slice 200 ultrafiltration system having a Hydrosart membrane with a molecular weight cut-off of 50 kDa (Sartorius). Finally, protein solutions were concentrated and diafiltrated against four volumes of phosphate buffer pH 8.3 using a centrifugal concentrator VIVACELL 100 (50 kDa cut-off; Sartorius) in a Beckman Allegra™ X-22R centrifuge, rotor SX4250 (Beckman-Coulter) at 3,900 × g.

### Vaccine formulation and analysis

Prior to adjuvation of the vaccine, protein solutions were adjusted to a concentration of 120 μg·ml^-1 ^and filtered through 0.45 and 0.22 μm cartridges (Sartorius AG) under sterile conditions in a laminar flow to obtain a sterile antigen solution. Adjuvation was made by mixing a solution of anhydromannitoletheroctodecenoate (Montanide ISA 50 V; Seppic, Paris, France) with the recombinant protein solution in batch-by-batch processes using a high-speed mixer Heidolph DIAX 900 (Heidolph Elektro, Kelheim, Germany) at 8,000 rpm and the vaccine was filled manually under sterile conditions in glass bottles of 20 ml (Wheaton, Millville, NJ, USA). Quality controls were made by testing mechanical and thermal stability of vaccine emulsions as described by Canales et al. [24].

### Rabbit immunization with recombinant proteins

Two New Zealand White rabbits per group was each immunized with 3 doses (weeks 0, 4 and 8) containing 50 μg/dose of purified recombinant proteins formulated as described above or Gavac (Revetmex, Mexico City, Mexico) as control. Rabbits were injected subcutaneously with 1 ml/dose using a 1 ml tuberculin syringe and a 27 1/2G needle. Two weeks after the last immunization, blood samples were collected from each rabbit into sterile tubes and maintained at 4°C until arrival at the laboratory. Serum was then separated after centrifugation and stored at -20°C. Rabbits were cared for in accordance with standards specified in the Guide for Care and Use of Laboratory Animals.

### SDS-PAGE, dot blot and Western blot analyses

Protein samples were analyzed by denaturing SDS-PAGE with a 12% PAGEgel-SDS cassette gel (PAGE-gel Inc, San Diego, CA, USA) under reducing conditions. Protein bands were visualized by either Coomassie Brilliant Blue R250 or silver staining. Samples were treated with dithiothreitol (DTT) reducer (PAGE-gel Inc.), heated in boiling water for 5 min before loading onto the gel and electrophoresed for 80 min at 90 mA constant current.

Electrophoretic transfer of proteins from gels to nitrocellulose membranes (PROTRAN BA85; Schleicher and Schuell, Dassel, Germany) for Western blot analysis was carried out in a Minie-Genie Electroblotter semi-dry transfer unit (Idea Scientific, Corvallis, OR, USA) according to manufacture's protocol. Protein samples of 2 μl were absorbed onto nitrocellulose membrane by gravity flow to perform the dot blot analysis. A standard curve was constructed with known amounts of recombinant Bm86 extracted from Gavac (Revetmex) and was used for semi-quantitative analysis in dot-blots. The supernatant of the GS115/Albumin strain (Invitrogen) grown under the same conditions was used as a negative control in both dot- and Western-blots. Membranes for dot or Western blots were blocked with 5% skim milk for 1 hr at room temperature, washed three times in TBS (25 mmol/L Tris·HCl, 150 mmol/L NaCl, pH 7.6) and probed with sera from rabbits immunized with Gavac (Revetmex) (1:1000 dilution) or recombinant proteins (1:5000 dilution) as described above. The antisera were diluted in 3% BSA in TBS. Membranes were then washed three times with TBS and incubated with an anti-rabbit IgG horseradish peroxidase (HRP) conjugate (Sigma-Aldrich) diluted 1:1000 in TBS. After washing the membranes again, color was developed using TMB stabilized substrate for HRP (Promega).

## Authors' contributions

MC carried out the expression, fermentation and protein purification and characterization studies. JMPL, VN and AMN carried out the genetic studies and participated in the sequence alignment. MH participated in the design of the study and helped to draft the manuscript. JF did the sequence alignment. FJ and JF conceived the study, and participated in its design and coordination and helped to draft the manuscript. All authors read and approved the final manuscript.
